# Global warming “heating up” the ICU through *Candida auris* infections: the climate changes theory

**DOI:** 10.1186/s13054-019-2702-4

**Published:** 2019-12-19

**Authors:** Giovanni Misseri, Mariachiara Ippolito, Andrea Cortegiani

**Affiliations:** Department of Surgical, Oncological and Oral Science (Di.Chir.On.S.). Section of Anesthesia, Analgesia, Intensive Care and Emergency. Policlinico Paolo Giaccone, University of Palermo, Via del vespro 129, 90127 Palermo, Italy

The simultaneous and independent worldwide outbreaks of *Candida auris* invasive infections seem to be a puzzling paradox [1, 2]. Since its first isolation, *C. auris* has risen several questions on how it could have appeared, survived, and thrived [1]. Several speculative hypotheses have been proposed. Although misuse of antimicrobials and over-abuse of azoles have been considered the main contributors to *C. auris* emergence [2, 3], these do not completely justify its spreading.

One of the most recent theories considers changes in climate conditions as a causative factor altering infectious disease ecology [4, 5] (Fig. 1). Humans and microbes had been influencing each other for decades. Global warming is one of the major components of climate change connected to human activities, having considerable impact on health and indirectly boosting infectious diseases. Only few fungal species can be considered as pathogenetic for humans, as the majority of mammals are remarkably resistant to invasive fungal diseases. Besides immunological responses, humans are characterized by a “thermal restriction zone” that protects against infections. Human-induced climate changes may be responsible for the progressive narrowing of this thermal restriction zone, defined as the difference between human basal temperature and environmental temperature. As *C. auris* is more thermotolerant if compared to other yeasts, global warming might have played an important role in its emergence [4]. Although the specific ecological niche has not been identified yet, the climatic oscillations effect on wetlands might have contributed to enrich this potential habitat, conferring thermal and salinity tolerance to *C. auris* non-pathogenetic naïve strains. Acquisition of virulence factors might be explained considering the potential transfer of virulence genes from other pathogenetic *Candida* spp. to *C. auris* naïve strains, or by the combination of global warming and UV radiations that might have induced genetic mutations. The upgrade of *C. auris* strains, from saprophyte to pathogenetic yeasts, has witnessed an intermediate avian host, thus permitting its transmission to humans. Overtime, genetic and epigenetic changes have led to an extreme adaptability of *C. auris* to different ecological niches, leading to the development of persistent outbreaks in healthcare settings [4, 5].

**Fig. 1:**
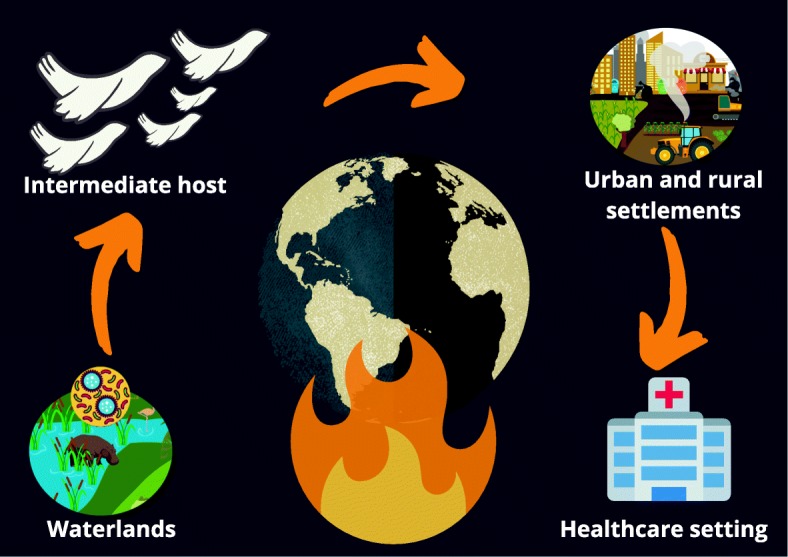
Global warming and the climate changes theory for *C. auris* emergence and spread. Rising ambient temperatures (caused by human activities) might have selected thermotolerant yeasts in wetlands; subsequently, acquiring opportunistic traits, *C. auris* might have spread through different ecosystems (wetlands, rural, and urban areas) thanks to intermediate avian hosts; following development of resistance and resilience through interspecies transmission, *C. auris* invades healthcare settings, leading to persistent outbreaks and causing infections in susceptible critically ill patients

Although global warming seems to be an appealing theory, it is not possible to ignore other factors which might explain *C. auris* rise. High population densities, poor hygiene, migrations, international travels, and pollution might indeed have contributed to the persistence of *C. auris* and acquisition of antifungal resistance [4]. Future studies are needed to identify its evolutionary reservoirs and validate the climate changes theory.

## Data Availability

Not applicable.

## Abbreviation

ICU: Intensive care unit
